# High satisfaction rate and range of motion can be expected in frozen shoulder after awake manipulation with brachial plexus block

**DOI:** 10.1186/s10195-024-00747-5

**Published:** 2024-01-28

**Authors:** F. Inglese, M. Montemagno, A. Brigo, M. Nigro, A. Giorgini, G. M. Micheloni, G. Porcellini

**Affiliations:** 1 Shoulder Team S.R.L., Forlì, Italy; 2 Department of General Surgery and Medical Surgical Specialties, Section of Orthopaedics and Traumatology, Hospital Policlinico–San Marco, University of Catania, Catania, Italy; 3 Orthopedic and Traumatology Department, University of Modena and Reggio Emilia, Modena, Italy; 4 Villa Maria Cecilia Hospital, Cotignola, Ravenna, Italy

**Keywords:** Adhesive capsulitis, Brachial plexus block, Passive mobilization, Physiotherapy, Treatment

## Abstract

**Background:**

Adhesive capsulitis (AC) is a disease of the glenohumeral joint that is characterized by pain and both passive and active global stiffness with a slow and insidious onset. The disease can occur spontaneously (primary AC) or it can be secondary to other comorbidities, surgery, or trauma, such as fracture or dislocation. Multiple treatment approaches have been suggested: intra-articular steroid injection, physical therapy, manipulation under total anesthesia, and arthroscopic or open surgery. Shoulder manipulation under anesthesia is usually proposed to patients that suffer from severe AC and have already undergone several nonoperative treatments without benefit. Different techniques have been proposed. This study presents our manipulation technique and the clinical results we achieved after shoulder mobilization under brachial plexus block in patients with phase III primary AC.

**Materials and methods:**

A retrospective cohort study was performed on a sample of 110 patients with phase III AC who were treated with this manipulation and followed up for 1 year. Patients underwent two assessments—before the procedure (T0) and 4 months after it (T1)—based on the Numerical Rating Scale, Simple Shoulder Test, and joint range of motion to assess shoulder pain, function, and joint articulation, respectively. Furthermore, the patients had to express their degree of satisfaction with the procedure and the results achieved.

**Results:**

Positive and statistically significant results were recorded in terms of pain reduction (ΔNPRS = − 5.4; *p* < 0.01) and improved functionality (Simple Shoulder Test Δ = 5; *p* < 0.01). Passive range of motion was statistically significantly increased for each movement at T1. Large increases were observed in extrarotation range of motion (ROM): R1 (Δ = 77.5°) and R2 (Δ = 70°), whereas little improvements were observed in intrarotation ROM. Patients achieved satisfying functional and articular recovery in all cases. Complications that needed further treatment occurred in three cases: a brachial plexus injury, a glenoid flake fracture, and persistent pain and stiffness.

**Conclusions:**

In this study, we proposed a standardized method of manipulation under brachial plexus block for patients affected by phase III adhesive capsulitis. The technique was applied among a large cohort of patients, who reported a high satisfaction rate and range-of-motion recovery after 4 months. This could represent an alternative treatment to surgery that has a shorter timeline and does not require patient hospitalization.

*Level of evidence*: Level III, retrospective cohort study.

## Introduction

Adhesive capsulitis (AC) is a glenohumeral disease that causes painful stiffness during both active and passive motion of the shoulder. The prevalence in the general population is around 3–5% but reaches 20% in diabetic patients. This disease was called “frozen shoulder” until 1945, when it was renamed AC to avoid the misleading term “frozen,” which was considered clinically unhelpful to describe the pathology.

By accurate physical examination, it is possible to observe decreased active and passive range of motion (ROM) of the shoulder, a loss of joint play, and consequently an increase in scapulothoracic compensation [1].

AC is classified into primary (or idiopathic) and secondary AC. Idiopathic AC occurs in the absence of a known inciting cause. It is associated with stressful events, strong emotions, or other conditions in which an underlying etiology cannot be identified. Secondary AC is attributable to a well-known traumatic inciting event or an associated pathology, such as thoracic surgery, shoulder fractures or arthritis, rotator cuff tears or tendinitis, or other clinical conditions.

Up to 70% of patients diagnosed with AC are women aged from 40 to 59 years old [2].

The most prevalent comorbidities in patients with AC are diabetes (30%) [3] and hypothyroidism (27.2%) [4]; moreover, patients with cardiovascular disease, cerebrovascular disease such as a subarachnoid hemorrhage, and breast cancer have an increased risk of developing AC [2].

From a clinical point of view, we can observe three phases of AC: (1) the freezing phase, in which patients complain of permanent shoulder pain, even at rest, and gradual limitation of shoulder movement; (2) the frozen phase, in which the main symptoms are articular stiffness in both active and passive ROM and a progressive reduction in pain; and (3) the thawing phase, which is defined by the resolution of pain, except for end-range movements where the capsular stiffness restricts the patient’s ROMs.

AC is usually described as self-limiting in 1–3 years, a period in which patients suffer great distress due to pain and  the loss of a normal routine of life. Indeed, up to 50% of patients are affected by long-lasting symptoms [2, 5, 6].

Several nonoperative treatments have been proposed for AC, but there is still no consensus on the best nonoperative flowchart. Physical therapy is strongly suggested after phase II of AC when the pain is becoming more tolerable, and it should be the first line of treatment even when used in combination with other therapies such as ultrasonics, transcutaneous electrical nerve stimulation, short-wave therapy, low-level laser therapy, and hydrotherapy.

Different types of manipulation to treat AC are described. The Maitland and Kaltenborn techniques are the most used passive treatments in patients affected by AC. The Maitland technique (MT) is performed with oscillatory movements with four grades of amplitude: grades I and II correspond to small oscillations which are able to train the mechanical receptor and help to reduce the generation of the pain stimulus; grade III implies a larger amplitude; and grade IV releases the joint stiffness since the oscillations are applied against the tissue resistance.

The Kaltenborn technique (KT), based on the concave-convex rule, is performed by a therapist who applies sustained capsular passive distraction with roll and glide passive movements. In grade I, minor distraction is used to control the pain; in grade II and grade III, higher stretching forces that are able to enhance the joint ROM are applied. Both techniques are effective at improving pain according to the Visual Analog Scale (VAS) and at increasing the range of motion in patients affected by AC. No evidence was found that one technique gives results superior to those provided by the other one [7].

Nonsteroidal anti-inflammatory drugs (NSAIDs) and oral corticosteroids do not significantly change the disease course of AC, but they are useful to relieve pain during physical therapy and phase I.

Interestingly, different studies have reported that pain and ROM improved after intra-articular corticosteroid or sodium hyaluronate injection [8]. Hydrodilatation, whole-body cryotherapy, and intra-articular injection of botulinum toxin type A have not afforded better results than intra-articular corticosteroid injection.

A recent randomized blinded study demonstrated that physical therapy is superior to oral corticosteroids in terms of gain in ROM and the reduction in pain in phase II of AC [9].

The management of AC becomes more complex in phase III, when corticosteroid injections and physical exercises usually yield poor outcomes.

Operative treatments are usually proposed to those patients with AC symptoms that do not improve with nonoperative management. The most practiced options are arthroscopic capsulotomy and mobilization under general anesthesia (MUA), while a few studies have described mobilization under interscalene brachial plexus block.

Arthroscopic capsulotomy consists of anterior–inferior and posterior–inferior capsular release. If performed with intra-articular injection and controlled manipulation, it improves pain and range of motion within 6 weeks in 80% of the patients treated [10].

During manipulation under general anesthesia, the operator stretches the capsular structures and adhesions above the limits of the thickened capsule. This technique has not been widely appreciated because of the intraprocedural risks of excessive capsular tears, humeral or glenoid fractures, brachial plexus injuries, and labral lesions [11].

The uncertain benefits of MUA and the significant risk of complications compared with those of other available treatments have raised some significant concerns about this procedure [2].

Roubal et al. described glenohumeral gliding manipulation following an interscalene brachial plexus block in patients with AC, highlighting the importance of keeping a short manipulative lever arm. The authors described a modified manipulative technique from Kaltenborn, observing that posterior manipulation increased the available excursion in both internal and external rotation. This observation leads to the conclusion that the increase in joint space and excursion occurred both anteriorly and posteriorly, in contrast to the concave-convex rule. Similarly, flexion and abduction increased after manipulating in an inferior direction [1].

In 1998, Placzek et al. described translational manipulation associated with brief corticosteroid therapy and postprocedural stretching exercises as the key to immediate increases in ROM and function, as they reduce the pain, discomfort, and disability time during the natural self-resolution process [12].

The new procedure offered many advantages compared to standard MUA: (1) it could be performed in an outpatient clinic and (2) it could even be performed in patients who could not undergo general anesthesia. Two therapists applied translational forces over the humeral head parallel to the glenoid. If the gentle and progressive joint stretching was not enough to regain passive ROM, the therapists performed high-velocity thrusts [1, 12].

The aim of our case series study was to prove the safety and the efficacy of standardized awake shoulder manipulation with brachial plexus block. We expect that patients treated with this technique will show increased ROM, reduced pain, and increased shoulder function.

## Materials and methods

This was a retrospective observational cohort study. Patients were enrolled between July 2020 and July 2022 in an Italian outpatient clinic specializing in shoulder disease. All the manipulations were performed by the same therapist, an expert in joint manipulation.

The inclusion criteria were as follows. The patient was (1) aged < 55 years and (2) had a diagnosis of symptomatic phase III primary AC with a severe loss of shoulder ROM (severe ROM limitation was considered to be stiffness and limitations of passive shoulder lateral rotation, abduction, and internal rotation of more than 50% compared with the opposite side for at least 3 months). (3) Clinical and radiological data [magnetic resonance imaging (MRI) and X-rays] were collected that could be used to exclude patients with rotator-cuff lesions, glenohumeral osteoarthritis, and bone or cartilage lesions. (4) Patients affected by osteoporosis, as confirmed by their bone mineral density (BMD), were excluded from the study, while patients with osteopenia (i.e., with a T-score between − 1.0 and − 2.5) were included [13]. (5) Patients treated with steroid therapy for comorbidities and those who could not undergo local block anesthesia were excluded from this study. (6) There was 1 year of follow-up.

All the patients were enrolled through informed consensus and consented to the collection and processing of personal data.

### Preliminary assessment

Patients underwent a general questionnaire in which we collected personal data and anamnestic information: their dominant limb, sports they used to play, comorbidities, a psychological assessment (depression, stress events, others), previous treatments, and time of pain onset.

Patients were further evaluated using the Numerical Pain Rating Scale (NPRS) and Simple Shoulder Test (SST).

The NPRS is a numerical scale from 0 to 10 in which 0 represents “no pain” and 10 represents “the worst pain ever.” The patient was asked to state the NPRS value for the pain suffered in four specific moments: during daily activities, at rest, during night-time, and after large and sudden movements [14].

The SST is scored on a multidimensional scale that corresponds to how the pain affects the loss of movement in and the strength and range of motion of the shoulder in daily life. The test is based on 12 items, each with a binary response, and the execution time is about 2 min [15].

The active ranges of motion of the affected limb and the opposite limb while standing were assessed by the physiotherapist with the goniometric technique in order to compare their progress. The participant was evaluated for shoulder elevation, abduction, external rotation with both the arm adducted and the arm abducted, and internal rotation.

To simplify the analysis, collected abduction and elevation ROM angles were categorized into numeric ranges (1: 0–30°; 2: 31–60°; 3: 61–90°; 4: 91–120°; 5: 121–150°; 6: 151–180°). Intrarotation levels were also collected and categorized into numeric ranges (1: thigh; 2: buttock; 3: lumbar-sacral joint; 4: L3; 5: T12; 6: T7).

Continuous data are shown as the mean and standard deviation. The *t*-test was used to compare NPRS scores, SST scores, and ROMs between the T0 and T1 evaluations. The selected threshold for statistical significance was *p* < 0.05.

The minimal clinically important difference (MCID) was calculated using the distribution-based method of a small effect size. All statistical analyses were performed using the SPSS version 24.0 statistical software (IBM Corp., Armonk, NY, USA).

### Procedure

The anesthetist performed ultrasound-guided interscalene brachial plexus block anesthesia in an outpatient clinic. After that, the shoulder was warmed up with a heating pad for 10 min in order to prepare the tissues for mobilization. All the procedures were performed by a single physical therapist. The operator performed an elongation of the retracted tissues through a specific progression of passive mobilizations in all three planes of movement of the glenohumeral joint, thus carrying out lysis of the capsular and extra-articular adhesions. Articular crepitus is produced by mobilization over the limits of the shrunken capsule, which is stretched and released. The manipulation consisted of glide and roll “swing” movements (the physiological movements of a healthy shoulder), avoiding translational movements, which can cause complications [16].

In the procedure used, the patient starts in the supine position on the table: the physiotherapist carries out a series of mobilizations up to an angle of 360° with a proximal solid grip. The proximal grip is used to prevent a long lever arm, which can produce shear forces on the bone, increasing the risk of fracture [17].

**Fig. 1:**
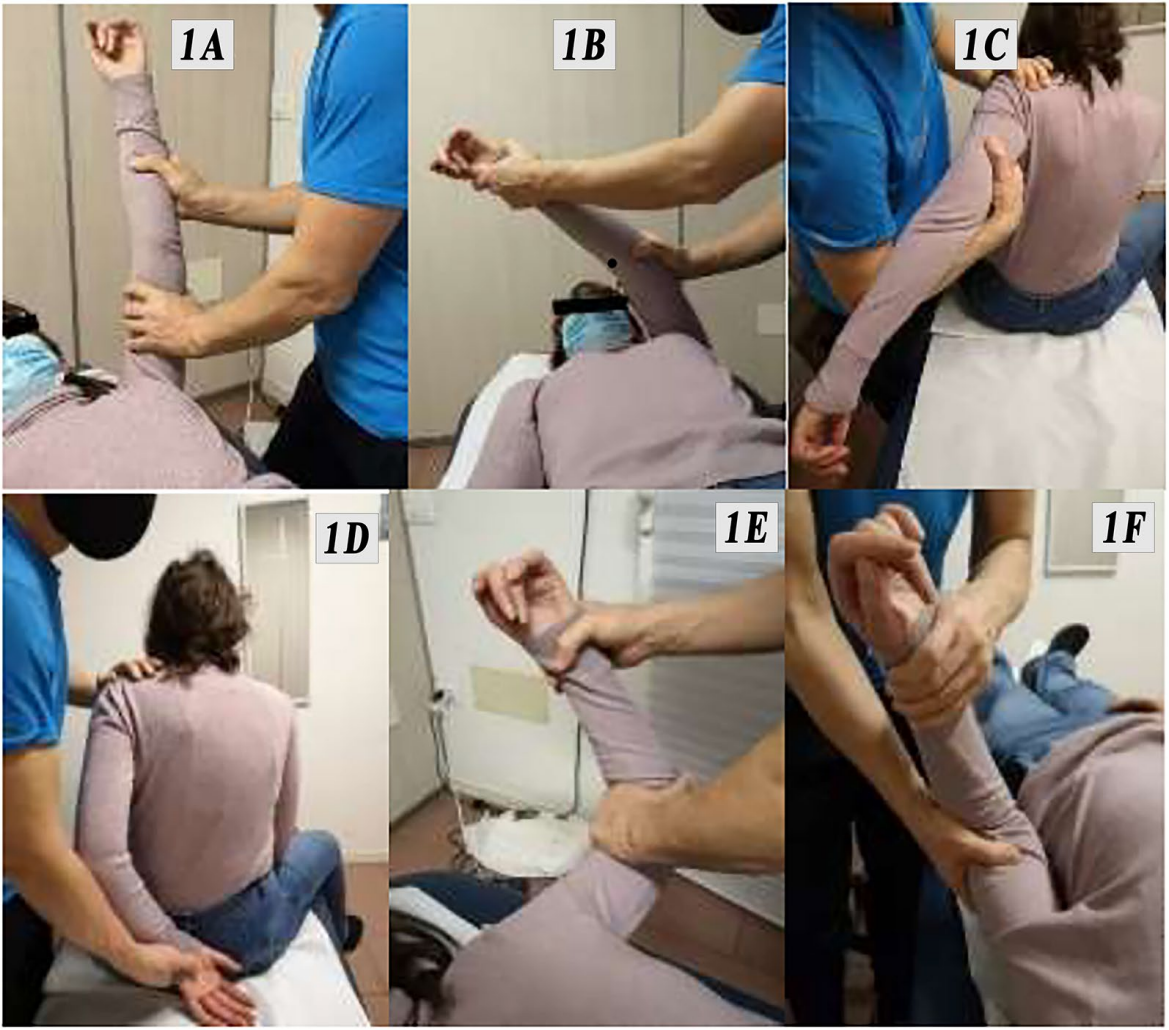
**A**–**F** Awake shoulder manipulation procedures performed by the physical therapist in order of timing

Similarly, in the first phase of the manipulation, rotational movements are avoided because the torsional forces generated can facilitate the onset of fracture in the presence of too many structured adhesions. Therefore, only single-plane movements are performed at the beginning. After the rupture of some adhesions, the joint backlash increases and the remaining capsular fibrosis breaks more easily: rotational movements are added from then on. The movements are performed in the following order:

1. Elevation to detach the posterior–inferior portion of the capsule (Fig. 1A).
2. Adduction in the horizontal plane to stretch the posterior portion of the capsule (Fig. 1B).
3. Extension (Fig. 1C) followed by internal rotation (Fig. 1D) to detach the posterior portion of the capsule. To perform these two movements, the patient is asked to position themselves sitting on the couch. This request is possible thanks to the type of anesthesia used and must be omitted in the case of mobilization performed under general anesthesia.
4. External rotation with the arm abducted to hold the anteroinferior portion of the capsule and extended to search for increased tissue tension (Fig. 1E).
5. External rotation with the arm adducted to hold the anterior–superior portion of the capsule (Fig. 1F).
6. Finally, the whole range of rotations is completed: external and internal.

The described sequence also implies an anatomical rationale: the anterior capsule has almost double the thickness of the posterior one because of ligament thickening and thus shows higher resistance to traction. In order to reduce possible complications, we first stress the less resistant capsular portion, gaining the mobility of the posterior capsule, so that stronger manipulation to recover the stiffer anterior capsule is subsequently possible.

The treatment ends when the physiotherapist perceives that the end of the line is soft in all directions of movement; there is no mechanical stop sensation, unlike before the manipulation. The procedure is completed with a clinical assessment of bone stability: the absence of crackles, a preternatural range of motion, and glenohumeral joint dislocation.

Patients were not administered oral steroid drugs or other painkillers. After the treatment, patients were encouraged to use local cryotherapy to manage the pain and the bruising of the shoulder. Finally, patients underwent shoulder X-rays to assess possible bone lesions.

### Complementary rehabilitation program

Once the joint mobilization was finished (after 10–20 min approximately), the patients were instructed and educated about the physiotherapy process that was necessary for the purpose of recovery. The rehabilitation program consisted of two aspects:

1. Passive mobilizations in all planes of movement, as performed by a physiotherapist three times per week for the first 4 weeks
2. Self-assisted treatment with active stretching exercises performed three times per day.

Every exercise was explained beforehand to each study participant.

**Fig. 2:**
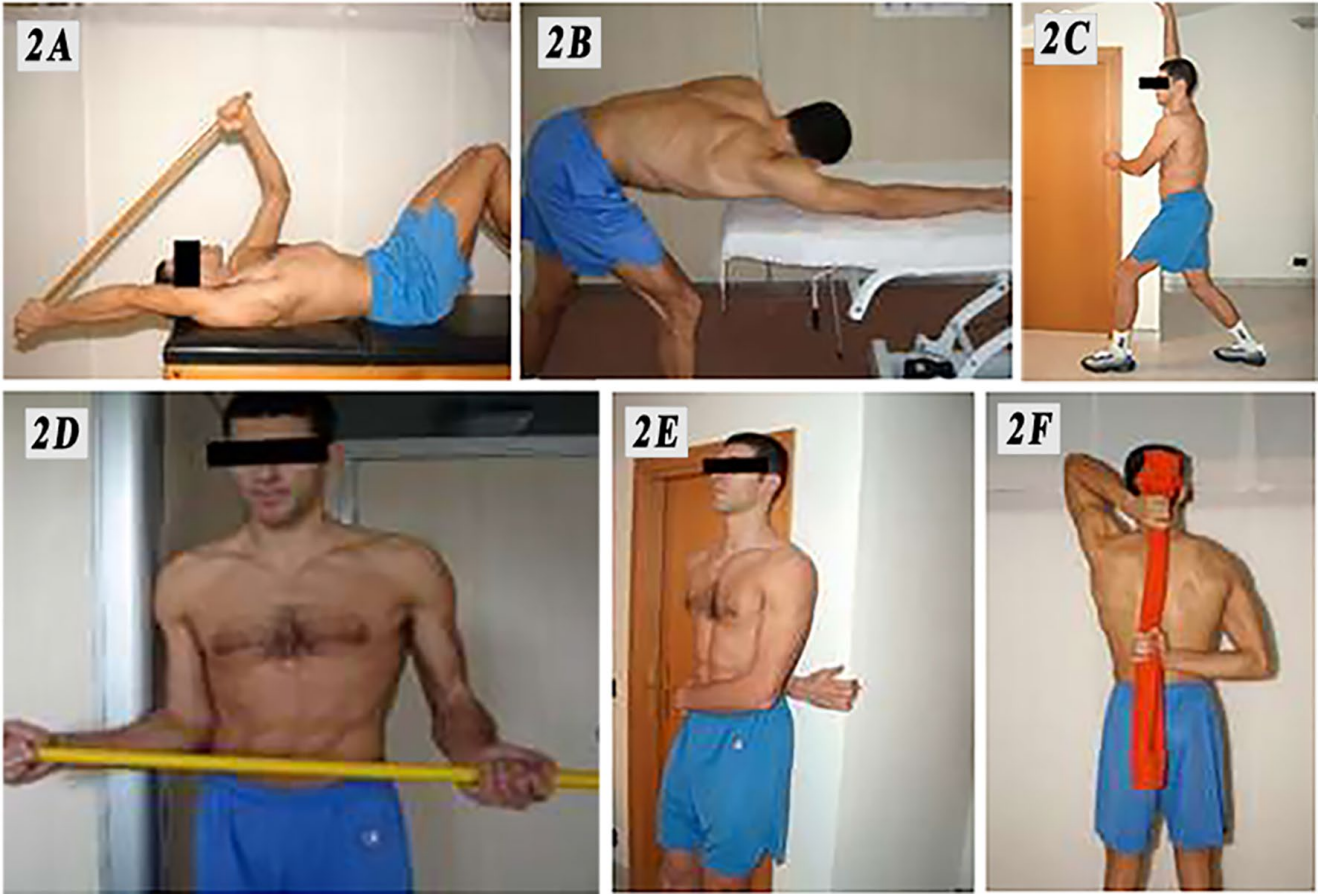
**A**–**F** Complementary home rehabilitation program assigned to all patients in the study

The exercises were explained to the patients as follows:

- Front elevation (Fig. 2A)

Assume a supine position with knees flexed and feet supported. With the help of a stick, and helping yourself with the healthy arm, elevate the affected arm as much as you can.

Variation: standing and facing a table, extend your arm by sliding your hand on the support surface (Fig. 2B).

- Anteposition with respect to the wall (Fig. 2C)

Standing, face the wall or a door jamb with one leg front and one back. Place the hand of the affected arm on the wall and slide it gradually upwards.

- External rotation of adducted arm (Fig. 2D)

Standing, with your side leaning onto the jamb of a door, place your arms along your side and bend your elbows at 90°. With the help of a stick, push the affected limb in external rotation without ever removing the elbow from your side.

- Internal rotation (Fig. 2E)

Stand with your back towards the wall. With the hand of the affected arm, hook onto the edge of the wall or the door jamb and move laterally (to the right for the right arm and the left for the left arm). After reaching the maximum lateral displacement possible, you can increase the rotation by bending the knees and gliding with your back to the wall.

Variation: stand up; with the help of a towel, drag the affected arm up along the back (Fig. 2F).

Patients were interviewed at two different times during the study: the first was before the treatment (T0) and the second was 4 months after the treatment (T1). In both evaluations, the NPRS and SST were applied to the patients and the ROM was evaluated with a goniometric technique by the same therapist who performed the previous measurement. Moreover, during the latter evaluation, the patient was asked to communicate their level of satisfaction.

## Results

One hundred ten subjects were enrolled in the study. The mean age was 45 years (range 36–55 years). The male:female ratio was 66/44, and 85 patients in total were diagnosed with primary AC (77.3%). Twelve patients showed bilateral AC. Thirty-seven patients (34%) were affected by diabetes or thyroid pathology. Other demographic features of the patient cohort are described in Table 1.

**Table 1:** Clinical and demographic features of the cohort of patients included in the study

Patient demographics	*N*	%
Age	110	Mean 45 (36–55)
Sex		
Male	66	60
Female	44	40
AC type		
Primary	85	77.3
Secondary	25	22.7
Painful shoulder		
Right	56	51
Left	54	49
Contralateral AC episode		
Yes	12	10.9
No	98	89.1
Associated disease		
Absent	73	66
Diabetes	10	9
Thyroid disease	13	11.8
Diabetes + thyroid	3	0.03
Pain duration		
< 3 months	12	10.9
3–6 months	20	18.1
6–12 months	47	42.7
> 12 months	31	28.1
Previous therapies		
None	8	7.3
Tecar, laser	61	55.5
Pharmacological	61	55.5
Manual rehab	84	76.4
Pool	24	21.8
Infiltrations	52	47.3
Other	18	16.4
Previous therapy efficacy		
Not performed	8	7.3
Nothing	35	31.8
Bad	33	30
Medium	31	28.2
Good	3	2.7
Psychological assessment		
Depression	22	20
Post-traumatic stress	*41*	*37*
Other condition	6	5

Outcome variables are expressed in Table 2. Comparing the T0 and T1 evaluations, there was a reduction in the NPRS (Δ = − 5.4; *p* < 0.01) and a gain in the Simple Shoulder Test (Δ = 5; *p* < 0.01).

**Table 2:** Clinical outcome variables (NPRS, SST, and ROM) measured in the study and their respective *p* values

Outcome		Mean	SD	Median	IQR	∆ Median post–pre	*p* value	MCID	% ∆ > MCID
NPRS T0		7	1.7	7	2.5	− 5.5	< 0.0001	0.47	96%
NPRS T1		1.9	1.1	1.5	1.5				
SST T0		4.2	1.9	4	4	6	< 0.0001	0.44	98%
SST T1		9.5	1	10	1				
ROM	Elevation T0	3.8	1	4	2	2	< 0.0001	0.40	95%
	Elevation T1	6	0	6	0				
	Abduction T0	3.25	0.97	3	1	3	< 0.0001	0.57	96%
	Abduction T1	6	0	6	0				
	Extrarotation R1–T0	14.3	16.2	10	20	80	< 0.0001	0.83	98%
	Extrarotation R1–T1	85.2	11.3	90	0				
	Extrarotation R2–T0	− 9.2	29.6	0	52.5	90	< 0.0001	0.45	92%
	Extrarotation R2–T1	81.3	16.1	90	0				
	Intrarotation T0	2.2	0.7	2	1	4	< 0.0001	0.85	99%
	Intrarotation T1	5.5	0.6	6	1				

*NPRS* Numerical Pain Rating Scale, *SST* Simple Shoulder Test, *ROM* range of motion, *R1* arm adducted, *R2* arm abducted 90°, *T0* test performed before the treatment, *T1* test performed 4 months after the treatment, *IQR Interquartile range,* *MCID* minimal clinically important difference

Regarding the ROM data expressed in the table, there was a statistically significant (*p* < 0.01) improvement in each movement at T1.

The MCID for each variable and the percentage of outcomes exceeding the MCID are indicated in Table 2.

### Complications

Three complications (2.7%) were recorded. A glenoid flake fracture occurred in a patient with previous humeral head plate fixation; this was treated conservatively and the patient recovered fully after 15 days.

Moreover, we observed a brachial plexus stupor characterized by residual paresthesia after the manipulation. In this case, the patient healed after rest and neurotrophic drug therapy in 2 months.

One patient showed pain and stiffness that persisted after treatment for 1 month. In this case, we decided to perform an arthroscopic capsular release. After the procedure, the patient recovered shoulder function.

## Discussion

This study accurately describes the steps in our manipulation method and strongly demonstrates, on a large cohort of patients, that it works as a treatment for adhesive capsulitis. It improves ROM, SST, and NPRS, has a low complication rate (2.7%), and does not require patient hospitalization.

Several treatments have been proposed to cure pain and stiffness in AC, but no consensus on the gold standard treatment has been achieved. Physical therapy, intra-articular injections of steroids, manipulations, and arthroscopic surgery alone have prompted some concern since none of them is considered the best resolutive therapy in phase III of AC. Many studies have suggested associating at least two techniques in severe forms of AC [18–20].

Moreover, patients affected by AC demand rapid ROM restoration and pain relief; for this reason, arthroscopic release is one of the most effective therapies, but it is still not the recovery therapy for AC: when arthroscopic surgery is associated with intra-articular injection of corticosteroids and physiotherapy, the treatment improves pain in over 50% of cases after 1 week, and 90% of patients experience good pain relief in 3 months. Almost 10% of patients continue to have pain despite the surgery. In the nondiabetic population, up to 79% of patients recover a forward flexion of greater than 160°, 73% regain internal rotation to L1, and 55% regain external rotation of greater than 70° [10].

In recent years, there has been increasing evidence supporting manipulation under anesthesia, which is described as a safe, reproducible, and efficient technique for shortening the period of shoulder stiffness [11].

Few studies state the limited and inconsistent evidence on the efficacy of this manipulation compared to other nonsurgical strategies [21].

The manipulation pattern depends on the clinician’s experience, and the manipulative steps are not always standardized: every clinician has their method, which is always associated with postmanipulation physical therapy. In previous studies, the Kaltenborn and Maitland techniques were applied with good results in terms of improving pain (VAS pre-KT was 5.58 ± 0.8 and VAS post-KT was 2.65 ± 0.67, whereas VAS pre-MT was 6.05 ± 1.12 and VAS post-MT was 3.12 ± 0.98) and increasing the range of motion in patients affected by AC (internal rotation: pre-KT 31.98 ± 6.17°, post-KT 37.32 ± 7.76°; external rotation: pre-KT 38.8 ± 5.75°, post-KT 49.64 ± 5.17°; internal rotation: pre-MT 31.74 ± 6.77°, post-MT 36.84 ± 6.90°; external rotation: pre-MT 40.85 ± 7.51°, post-MT 49.76 ± 8.64°) [7].

Recently, many clinicians have explored the mobilization technique throughout the shoulder’s range of motion by first applying anterior elevation stress and then with external rotation, internal rotation, and abduction [22, 23]. Some clinicians prefer a supine position for the patient under general anesthesia [11, 22], whereas some others share our preference for collaborating with the awake patient under brachial plexus block [12, 23].

The translational technique explored by Placzek et al. brought notable ROM recovery results: at discharge, flexion was 154° (145–163°), abduction was 156° (144–168°), internal rotation was 58° (53–63°), and external rotation was 76° (69–83°). We avoided those manipulation techniques to avoid iatrogenic fractures and an unwanted instability of the glenohumeral joint, which can lead to wear of the articular surfaces in the long term. Tsvieli et al. explored Codman’s paradox in their manipulation under general anesthesia, obtaining good results in terms of pain relief and restitution of shoulder ROM: elevation and abduction increased to 177° and 175°, respectively; external rotation improved after the treatment from 6 to 76°; and internal rotation improved from 12 to 62° [16].

Our manipulation technique is quite innovative, and many details are described here for the first time: (1) the physiological movements are used to recover the articular range of motion; (2) this is a single-operator technique; (3) thrusts and high-velocity manipulations were avoided; (4) the proximal grip was used to prevent a long lever arm, which could produce rotational forces on the bone and increase the risk of fracture; (5) the patient is awake under peripheral anesthetic block and has to collaborate with the therapist during the manipulations by positioning the body in the most functional way for the action of the therapist.

Another positive aspect of our data is the number of enrolled patients, which is unusually high for this kind of study.

The results of the physical manipulation we described in this study are similar to the results of the arthroscopic surgical release technique, but with the benefit for the patient of shorter timelines and outpatient procedures.

The data we collected suggest that an accurate manipulation treatment associated with a post-treatment rehabilitation program could solve most AC cases; it presents a low risk and yields a high satisfaction rate of patients in a brief time.

In our opinion, this study strengthens the knowledge about manipulation techniques for AC and lays the foundations for a consensus flowchart that will help in the treatment of patients affected by AC.

This study has many limitations. First of all, it is a retrospective study and we have not selected a control group which could have helped to strengthen our results. The 1-month and 4-month outcomes could have been enhanced with a longer follow-up in the following year.

Another limitation is that no patients were checked using arthroscopy postmanipulation to assess iatrogenic damage.

Sasanuma et al. investigated the soft-tissue lesions associated with manipulation under a cervical nerve root block and found a large number of soft-tissue lesions (mid-substance lesions or the humeral avulsion of a glenohumeral ligament, anterior labrum tears, bone bruises) by comparing post-treatment MRI results with pretreatment MRI results [23].

Similarly, Loew et al. investigated the lesions present after manipulation under general anesthesia with an arthroscopic view, and they observed several undesired articular lesions, such as some labral detachment ruptures of a glenohumeral ligament and osteochondral lesions. These lesions were detected by the high sensitivity of the MRI and the arthroscopic view, but the clinical correlations are still debatable, since the improvement in shoulder ROM in all planes after the procedure was evident [11].

Some authors described a lower complication rate (< 0.2%) for the MUA procedure compared to our method [22], although some patients have persistent or recurrent symptoms requiring another MUA of the shoulder [24].

Even though our manipulation technique is not the same as that used by our colleagues, we cannot exclude the possibility that the patients in our study reported some subclinical soft-tissue lesions aside from the complications that we have already discussed.

The clinical and scientific relevance of the awake shoulder manipulation proposed in this study is that it represents a quick, decisive, and nonsurgical treatment for phase III patients which resolves pain and increases shoulder mobility in an outpatient setting; it is also a relatively fast method compared to surgical treatments, pharmacological treatments, or long rehabilitations proposed in the literature.

## Conclusion

In this study, we presented a novel awake shoulder manipulation performed under brachial plexus block for patients affected by phase III adhesive capsulitis. The technique was applied among a large cohort of patients, who reported a high satisfaction rate, good range-of-motion recovery, and a low incidence of complications after 4 months. This could represent an alternative treatment to surgery that provides a shorter timeline and does not require hospitalization of patients.

## Data Availability

The datasets used during the current study are available from the corresponding author on reasonable request.

## Abbreviations

AC: Adhesive capsulitis
MT: Maitland technique
KT: Kaltenborn technique
ROM: Range of motion
ICAM-1: Intercellular adhesion molecule 1
NSAIDs: Nonsteroidal anti-inflammatory drugs
MUA: Mobilization under anesthesia
MRI: Magnetic resonance imaging
BMD: Bone mineral density
NPRS: Numerical Pain Rating Scale
SST: Simple Shoulder Test
MCID: Minimal clinically important difference
